# Effects of New Media–Based Education on the Treatment of Helicobacter pylori Infection: Systematic Review and Meta-Analysis

**DOI:** 10.2196/78387

**Published:** 2025-10-23

**Authors:** Wentao Fan, Yuwen Tao, Jinjin Shi, Feng Ye

**Affiliations:** 1 Department of Gastroenterology The First Affiliated Hospital with Nanjing Medical University Nanjing China; 2 Department of Gastroenterology The Fourth Affiliated Hospital of Nanjing Medical University Nanjing China

**Keywords:** Helicobacter pylori, H. pylori, new media education, eradication rates, patient compliance, adverse events, patient satisfaction

## Abstract

**Background:**

*Helicobacter pylori* (*H. pylori*) infection continues to pose a substantial global health burden. The eradication of *H*. *pylori* has been shown to substantially reduce the risk of gastric cancer. However, despite the availability of effective antimicrobial regimens, eradication rates remain suboptimal, largely due to poor patient adherence to treatment. The emergence of new media–based education (NME) offers an effective approach to enhancing patient understanding, engagement, and adherence, thereby improving the overall management of *H. pylori* treatment and follow-up care.

**Objective:**

This meta-analysis aimed to assess the impact of NME interventions on *H*. *pylori* eradication rates, patient compliance, adverse events, and patient satisfaction.

**Methods:**

We systematically searched PubMed, Embase, Web of Science, and the Cochrane Library from inception to February 2025. Eligible studies included randomized controlled trials (RCTs) and retrospective cohort studies comparing NME (eg, mobile apps, SMS text messages, and WeChat) with conventional education in patients with *H*. *pylori* infection. Studies were required to report at least one of the following outcomes: eradication rate, patient compliance, adverse events, and patient satisfaction. Two reviewers independently performed study selection, data extraction, and risk-of-bias assessment. Random-effects models were used to calculate pooled risk ratios (RRs) with 95% CIs. Subgroup analyses were conducted based on intervention type, therapy regimen, regional socioeconomic status, age group, and WeChat communication format. Sensitivity analyses tested the robustness of the pooled results.

**Results:**

A total of 13 studies (n=11, 85% RCTs and n=2, 15% retrospective cohort studies) involving 2942 patients were included. Overall, NME significantly improved *H*. *pylori* eradication compared with conventional education (81.9% vs 67%; RR 1.22, 95% CI 1.11-1.33; *P*<.001). Subgroup analyses showed greater benefits in patients receiving quadruple therapy (*P*<.001), those aged ≤60 years (*P*=.03), populations from low- and middle-income countries (*P*<.001), and 14-day regimens (*P*<.001). Notably, WeChat-based interventions (*P*=.002), especially one-on-one education (*P*=.006), produced the most pronounced effects, whereas telephone- and messaging-based methods showed limited impact. Patient compliance was also significantly higher in the NME group (90.5% vs 73%; RR 1.27, 95% CI 1.15-1.40; *P*<.001), particularly among younger patients (*P*=.03) and in 14-day regimens (*P*<.001). In contrast, NME did not reduce adverse events (*P*=.23). Patient satisfaction, reported in 23% (3/13) of the studies, was consistently higher with NME (*P*<.001). Most RCTs (7/11, 64%) were judged to be at low risk of bias, and sensitivity analyses confirmed the robustness of all primary outcomes.

**Conclusions:**

NME significantly enhances *H*. *pylori* eradication and patient compliance without increased adverse events. Personalized and interactive communication platforms, especially WeChat-based interventions, show substantial promise. These findings support the integration of tailored digital education into clinical practice, particularly in resource-limited or technology-adopting settings. Further high-quality RCTs are warranted to validate long-term efficacy and generalizability.

**Trial Registration:**

PROSPERO CRD42024517954; https://www.crd.york.ac.uk/PROSPERO/view/CRD42024517954

## Introduction

### Background

*Helicobacter pylori* (*H*. *pylori*) infections correlate with chronic active gastritis, peptic ulcer development, gastric mucosa-associated lymphoid tissue lymphoma, gastric cancer, and other digestive diseases, as well as extradigestive disorders such as cardiovascular, hematologic, and autoimmune diseases [1-3]. A recent meta-analysis of data from 73 countries revealed a global *H*. *pylori* infection rate of 44.3%, higher in low- and middle-income countries (50.8%) than in high-income countries (34.7%), imposing significant economic burdens [4]. These findings underscore *H*. *pylori*’s pervasive harm, heightening global public health concerns for its eradication [5]. Standard triple and quadruple therapy combining proton pump inhibitors or bismuth with 2 antibiotics is established for *H*. *pylori* eradication [6,7]. However, despite widespread antibiotic use, eradication rates remain suboptimal, often due to antibiotic resistance and poor patient compliance [8].

Poor patient compliance significantly impacts *H*. *pylori* eradication treatment as it increases the risk of treatment failure and contributes to the development of secondary antibiotic resistance due to inadequate drug dosages [9,10]. According to research involving a large patient cohort, those with good compliance, defined as completing over 80% of prescribed medication regimens, achieved eradication rates of 85% to 94%, whereas those with poor compliance achieved rates of only 39% to 53% [11]. Various factors influence patient compliance, including the complexity of the eradication regimen, treatment duration, adverse reactions, physician motivation, and patient awareness [10,12]. Currently, the prevailing eradication regimen typically lasts 7 to 14 days and involves administering 3 to 4 drugs before or after meals [13]. However, the regimen’s complexity and extended duration pose challenges for both physicians and patients, prompting widespread discussion among gastroenterologists on how to effectively address this issue.

### Objectives

To combat poor patient compliance in *H*. *pylori* eradication treatment, various strategies have been implemented to enhance medication adherence, including health education, information communication, medication guidance, feedback on adverse drug reactions, and regular question-and-answer sessions. The emergence of new media technologies—such as telephone-supported follow-ups, SMS text messaging, and social media– or app-based modules—has opened up new avenues for patient education and supervision. In this review, we define new media–based education (NME) as a form of patient education and adherence support distinct from traditional oral or paper-based approaches. It is digitally delivered and interactive, implemented through mobile or networked platforms (eg, telephone-supported follow-ups, SMS text messaging, and various social media– or software-based applications). NME typically incorporates targeted communication and reminders, multimedia educational content, and bidirectional messaging in alignment with established digital health (mobile health) frameworks [14-16]. Despite its widespread use, the precise impact of NME on *H*. *pylori* eradication remains uncertain. Three meta-analyses published in 2022 suggested that enhanced educational interventions may improve eradication rates and patient compliance without increased adverse events [17-19]. However, these reviews were limited by relatively small sample sizes and the absence of comprehensive subgroup analyses, leaving uncertainties regarding the consistency and generalizability of their findings. To address these gaps, we conducted an updated and more comprehensive meta-analysis to evaluate the efficacy of NME in improving *H*. *pylori* treatment outcomes.

## Methods

### Registration and Implementing Guidance

This study adhered to the PRISMA (Preferred Reporting Items for Systematic Reviews and Meta-Analyses) guidelines and was registered on PROSPERO under registration number CRD42024517954.

### Search Strategy

We systematically searched PubMed, Embase, Web of Science, and the Cochrane Library from inception to February 2025. The review protocol was registered in PROSPERO in February 2024, and an updated search was conducted in February 2025 to ensure the inclusion of the most recent studies. Eligible study designs included randomized controlled trials (RCTs) and retrospective cohort studies evaluating NME interventions for patients with *H*. *pylori* infection. The search strategy combined MeSH (Medical Subject Headings) terms and free-text keywords such as “*Helicobacter pylori*,” “patient education,” “educational technology,” “mobile phone applications,” “smartphone,” “short message service (SMS),” “text messaging,” “WeChat,” “tele-education,” “internet,” and “telemedicine” using Boolean operators (AND and OR) to maximize sensitivity. An example search string for PubMed was as follows: (“Helicobacter pylori” OR “H. pylori”) AND (“patient education” OR “health education” OR “educational technology” OR “mobile phone” OR “cell phone” OR “smartphone” OR “text message*” OR “short message service” OR “SMS” OR “WeChat” OR “tele-education” OR “telemedicine” OR “digital health”) AND (“randomized controlled trial” OR “controlled clinical trial” OR “cohort study”). The reference lists of the included studies and relevant reviews were also screened to identify additional eligible articles. The complete and detailed search strategies for each database are provided in Multimedia Appendix 1 in accordance with the PRISMA 2020 reporting guidelines.

### Study Selection

Two investigators independently screened the initially retrieved bibliography using a 2-stage screening process. Any discrepancies were resolved through discussion or consultation with a third investigator. In the first phase, duplicates were automatically removed, followed by manual removal of any remaining duplicates. Studies were excluded based on inclusion and exclusion criteria by reviewing titles, abstracts, and full texts.

The following inclusion criteria were used in this study: (1) participants were individuals with *H*. *pylori* infection confirmed via rapid urease test, urea breath test, stool antigen test, culture, or histology with at least one positive result and who had not previously received eradication therapy; (2) the intervention was implementation of NME methods, such as telephone-based follow-up, SMS text messaging, or mobile apps, in combination with a standard eradication regimen (triple or quadruple therapy); (3) the comparator was a standard eradication regimen with conventional oral or written instructions; (4) the primary outcomes included *H*. *pylori* eradication rate and patient compliance, and the secondary outcomes included adverse events and patient satisfaction; and (5) the study design was RCTs and retrospective cohort studies. Retrospective studies were included due to limited available evidence and their comparable design and intervention quality to those of RCTs. The exclusion criteria were as follows: (1) publications in the form of comments, letters, protocols, conference abstracts, or reviews; (2) studies with insufficient data or duplicate reports; and (3) ongoing studies without available results.

### Data Extraction

Two independent investigators extracted data from eligible studies using a predefined data collection form. Discrepancies were resolved through consensus. Extracted data included study characteristics, patient demographics, outcomes, and details of risk-of-bias assessment.

### Risk-of-Bias Assessment

The methodological quality of the included studies was independently assessed by 2 reviewers, with disagreements resolved by a third reviewer. For RCTs, we applied the revised Cochrane risk-of-bias tool for randomized trials (RoB 2), which evaluates 5 domains: bias arising from the randomization process, bias due to deviations from the intended intervention, bias due to missing outcome data, bias in measurement of the outcome, and bias in the selection of the reported results. Each domain was judged as “low risk,” “some concerns,” or “high risk,” and an overall risk-of-bias judgment was assigned accordingly. In total, 11 RCTs were evaluated using the RoB 2 tool.

For nonrandomized cohort studies, we used the Risk of Bias in Nonrandomized Studies of Interventions (ROBINS-I) tool, which assesses 7 domains: bias due to confounding, bias in the selection of participants, bias in classification of interventions, bias due to deviations from the intended intervention, bias due to missing data, bias in measurement of outcomes, and bias in the selection of the reported results. The overall risk of bias for cohort studies was categorized as low, moderate, serious, or critical. The 2 retrospective cohort studies included in this review were assessed using the ROBINS-I tool.

### Statistical Analysis

We calculated risk ratios (RRs) and 95% CIs to evaluate associations between groups and outcomes. The primary outcomes were the pooled RRs of *H*. *pylori* eradication rate and patient compliance, whereas the secondary outcomes were pooled RRs of adverse events and patient satisfaction. We selected intention-to-treat (ITT) analysis as the primary approach because of its stronger scientific rationale, greater clinical applicability, and its ability to reflect real-world practice. Although some of the included studies (10/13, 77%) reported per-protocol (PP) data, only 69% (9/13) provided PP results for eradication, and just 31% (4/13) provided PP results for compliance. Given this limited number, it was not feasible to conduct meaningful subgroup analyses based on PP data, and conducting a pooled PP analysis under such circumstances would have resulted in too small a sample size, thereby reducing statistical robustness and potentially introducing bias. Consequently, we relied exclusively on ITT analysis to ensure the consistency, validity, and reliability of the findings while also minimizing the risk of selective reporting.

Heterogeneity was assessed using the *I*^2^ statistic, with thresholds of 25%, 50%, and 75% representing low, moderate, and high heterogeneity, respectively. In line with current recommendations, we used a random-effects model for all meta-analyses to account for potential between-study heterogeneity and provide more conservative estimates.

Prespecified subgroup analyses were conducted to explore potential sources of heterogeneity. Subgroups were stratified according to intervention type (WeChat, telephone, telephone combined with other methods, and SMS text messaging), treatment regimen (triple vs quadruple therapy), age group (≤60 vs >60 years), country socioeconomic status (high income vs low and middle income), whether baseline outpatient education was provided, and whether WeChat group functionality was used.

Sensitivity analyses were conducted by sequentially excluding each study to assess its influence on the overall effect size.

Compliance definitions varied among studies, with 100% compliance defined as taking the prescribed dose. Publication bias was assessed using funnel plots, the Egger linear regression test, and the Begg rank correlation test. All primary meta-analyses were conducted using RevMan (version 5.4.1; The Cochrane Collaboration). Additional statistical analyses, including sensitivity analyses and publication bias assessments, were conducted using the Comprehensive Meta-Analysis software (version 3.0; Biostat, Inc).

## Results

### Study Flow

**Figure 1:**
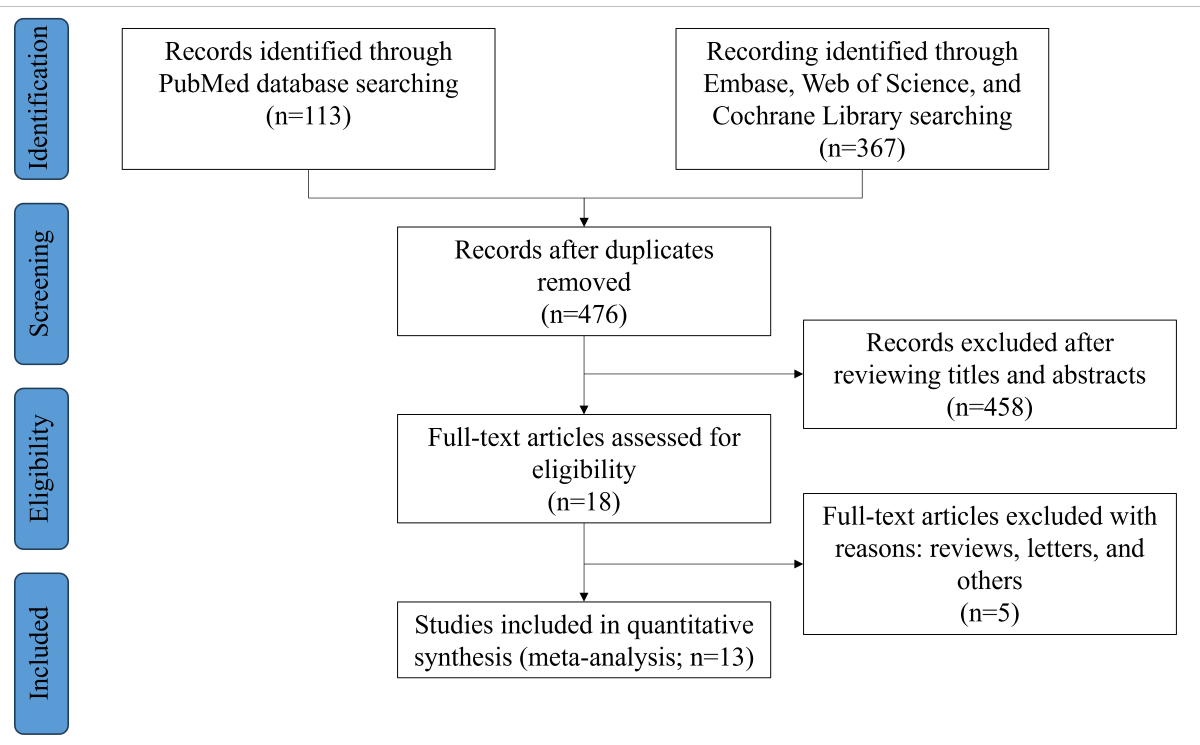
Flowchart of study selection.

Our initial search identified 476 potentially relevant clinical trials. After thorough review and screening, of the 476 studies, 11 (2.3%) RCTs and 2 (0.4%) retrospective cohort studies [20-32] met the inclusion criteria and were included in the meta-analysis. The study selection process is detailed in Figure 1.

### Risk of Bias

Using the RoB 2 tool [33], most of the 11 (85%) included RCTs were judged to be at low risk of bias, with some concerns mainly related to the randomization process and missing outcome data; no trial was rated as high risk (Figure S1A in Multimedia Appendix 2). The 2 retrospective cohort studies were assessed using the ROBINS-I tool [34], which indicated a moderate risk of bias for one study [22] and a serious risk for another [27], largely due to confounding and limitations in participant selection and outcome measurement (Figure S1B in Multimedia Appendix 2).

### Study Characteristics

The basic characteristics of the 13 eligible studies [20-32] are summarized in Table 1. These trials, conducted between 1999 and 2023, had sample sizes ranging from 80 to 566 participants, totaling 2942 across all studies. In total, 23% (3/13) of the trials [30-32] were conducted in high-income countries (England, the United States, and Australia), whereas the remaining 77% (10/13) [20-29] were conducted in low- and middle-income countries (China and Jordan). A total of 92% (12/13) of the studies [20-30,32] reported on the eradication rate of *H*. *pylori*, 100% (13/13) [20-32] reported on patient compliance, 69% (9/13) [20,21,23,25,27-30,32] reported on adverse events, and 23% (3/13) [23,28,29] reported on patient satisfaction. The *H*. *pylori* eradication regimens included triple and quadruple therapies, with durations ranging from 7 to 14 days (Table 1). Control group patients received traditional oral and written medical instructions, whereas experimental group patients received additional intensive education instructions.

**Table 1:** Characteristics of the studies included in this meta-analysis.

Study	Year	Country	Type	Sample size, n	Age (y), mean	Female/male participants, n	NME^a^ method	Eradication regimen	Duration	Detection
Al-Eidan et al [30]	2002	England	Retrospective cohort study	80	50	22/54	TEL+^b^	Triple therapy	7 d	RUT^c^
Shoiab et al [26]	2023	Jordan	RCT^d^	200	—^e^	97/103	TEL+	Triple therapy	14 d	SAT^f^
Lin et al [25]	2022	China	Retrospective cohort study	533	45	232/301	WeChat	Quadruple therapy	14 d	^13^C-UBT^g^
Wang et al [29]	2015	China	RCT	140	44	88/53	TEL^h^	Triple therapy	10 d	^13^C-UBT
Henry and Batey [32]	1999	Australia	RCT	119	57	50/69	TEL+	Triple therapy	10 d	^13^C-UBT
Ma et al [24]	2021	China	RCT	566	—	—	WeChat	Quadruple therapy	14 d	^14^C-UBT^i^
Sun et al [23]	2022	China	RCT	226	44	128/98	WeChat	Quadruple therapy	14 d	UBT^j^
Lee et al [31]	1999	United States	RCT	125	49	90/35	TEL+	Triple therapy	14 d	—
Luo et al [22]	2020	China	RCT	222	50	134/88	WeChat	—	14 d	^14^C-UBT
Wang et al [28]	2019	China	RCT	310	46	148/162	SMS text messaging	Quadruple therapy	14 d	^13^C-UBT
Zhao et al [27]	2020	China	RCT	162	45	92/70	TEL+	Quadruple therapy	14 d	^13^C-UBT
Chen et al [20]	2021	China	RCT	196	44	91/105	TEL	Quadruple therapy	14 d	^13^C-UBT
Yang et al [21]	2023	China	RCT	254	35	133/121	WeChat	Quadruple therapy	14 d	^13^C-UBT

^a^NME: new media–based education.

^b^TEL+: telephone combined with other methods.

^c^RUT: rapid urease test.

^d^RCT: randomized controlled trial.

^e^Not available.

^f^SAT: stool antigen test.

^g^C-UBT: carbon-13 urea breath test.

^h^TEL: telephone.

^i^C-UBT: carbon-14 urea breath test.

^j^UBT: urea breath test.

### Primary Outcome

#### H. pylori Eradication Rate

**Figure 2:**
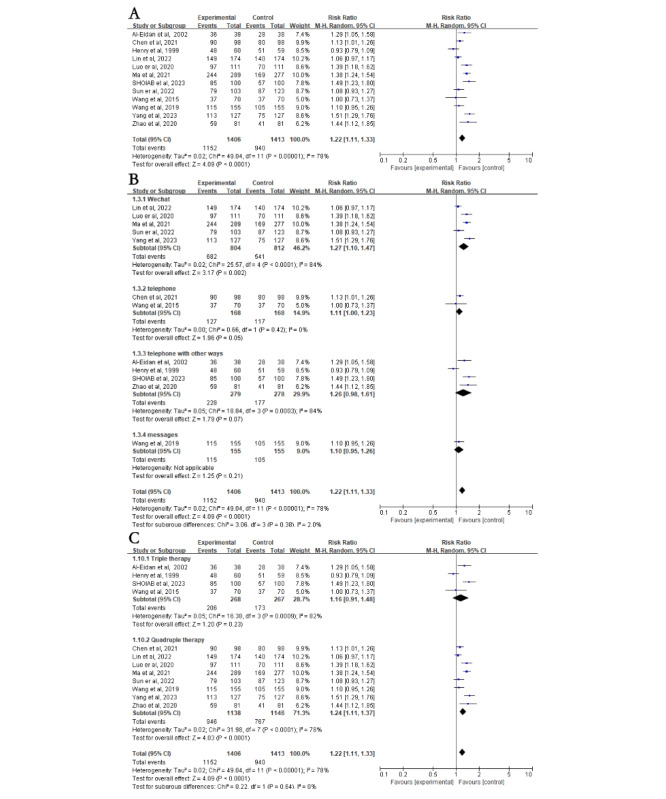
Forest plot comparing Helicobacter pylori (H. pylori) eradication in education versus control groups—subgroup analysis on intervention methods and regimens. (A) Forest plot comparing the H. pylori eradication rate between the education and control groups. (B) Subgroup analysis for H. pylori eradication rates based on different intervention methods. (C) Subgroup analysis for H. pylori eradication rates based on different treatment regimens. Control: traditional instructions; experimental: new media–based education instructions; fixed: fixed-effects model; random: random-effects model.

A total of 92% (12/13) of the studies involving 2819 patients reported data on patient *H*. *pylori* eradication rates. ITT analysis showed that eradication rates were significantly higher in the NME group compared with the control group (81.9% vs 67%; RR 1.22, 95% CI 1.11-1.33; *P*<.001; Figure 2A [20-32]). A high level of heterogeneity was detected among the included studies (*I*^2^=78%; *P*<.001), but sensitivity analysis confirmed the stability of the results (Figure 2A).

#### Subgroup Analysis of H. pylori Eradication Rate

Heterogeneous results require subgroup analysis. Subgroup analysis included different new media devices, different medication regimens, different age groups, the socioeconomic status of the country of origin, whether initial outpatient education was provided, and whether WeChat group functionality was used in conjunction with WeChat. In the intervention method subgroup, patients were categorized into WeChat, telephone, telephone with other methods, and SMS text messaging. In the medication regimen subgroup, patients were categorized into quadruple therapy and triple therapy. In the subgroup analysis of different populations, individuals were divided into age groups of ≤60 and >60 years and into high-income and low- and middle-income country groups based on the country of origin.

**Figure 3:**
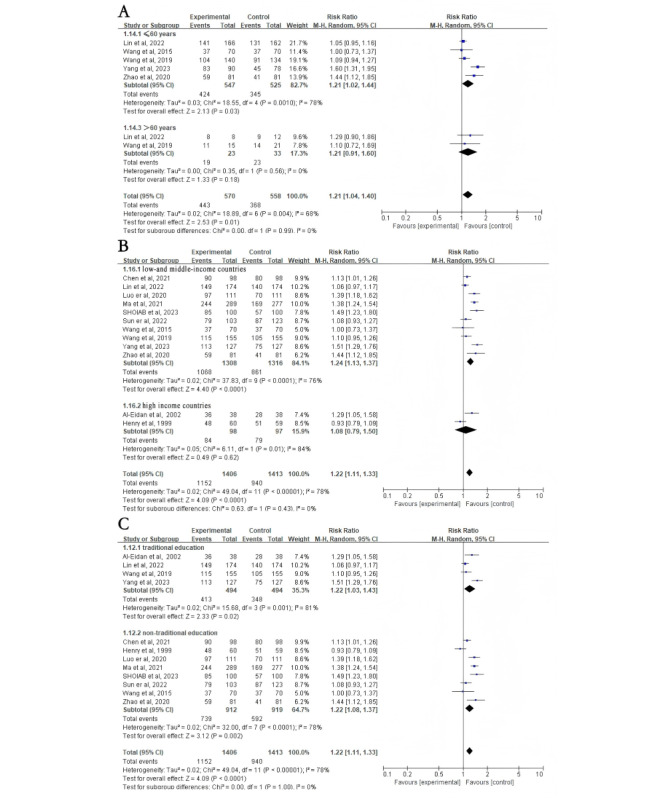
Subgroup analysis on Helicobacter pylori (H. pylori) eradication rates based on age (>60 years), country socioeconomic status (high income vs low and middle income), and education (traditional). (A) Subgroup analysis for H. pylori eradication rates based on whether patients were older than 60 years. (B) Subgroup analysis for H. pylori eradication rates based on whether patients were from high-income or low- and middle-income countries. (C) Subgroup analysis for H. pylori eradication rates based on whether patients received traditional education. Control: traditional instructions; experimental: new media–based education instructions.

**Figure 4:**
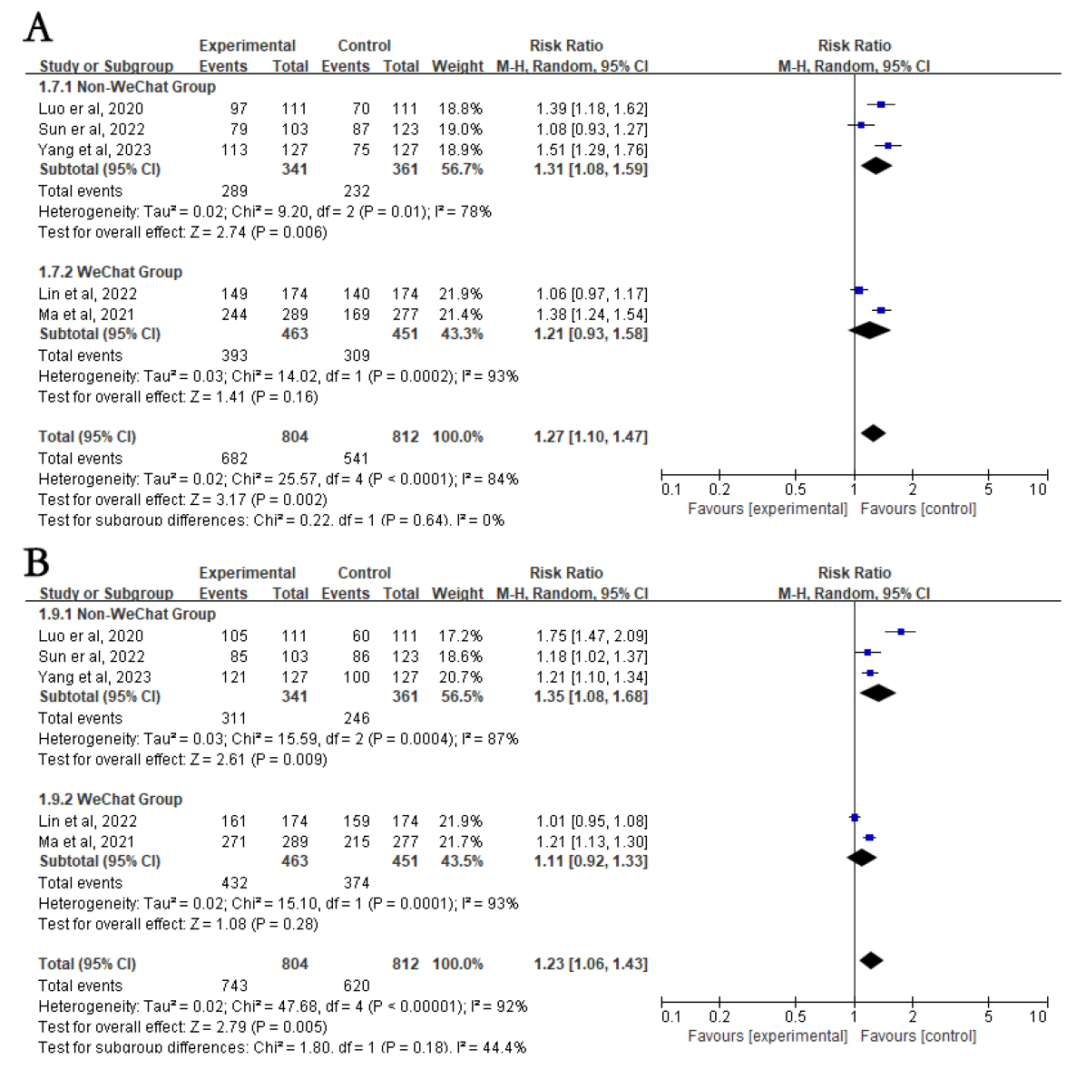
Subgroup analysis of Helicobacter pylori eradication rates and patient compliance based on whether patients used WeChat groups. (A) Subgroup analysis for Helicobacter pylori eradication rate. (B) Subgroup analysis for patient compliance. Control: traditional instructions; experimental: new media–based education instructions.

First, subgroup analyses were stratified according to different intervention methods. The intervention group had higher eradication rates in the WeChat subgroup (RR 1.27, 95% CI 1.10-1.47; *P*=.002; Figure 2B). However, there was no difference in eradication rates between the intervention and control groups in the telephone (RR 1.11, 95% CI 1.00-1.23; *P*=.05), telephone with other methods (RR 1.26, 95% CI 0.98-1.61; *P*=.07), and SMS text messaging (RR 1.10, 95% CI 0.95-1.26; *P*=.21; Figure 2B) subgroups. Subgroup analyses were then stratified according to different medication regimens. The intervention group had higher eradication rates than the control group in the quadruple therapy subgroup (RR 1.24, 95% CI 1.11-1.37; *P*<.001) but not in the triple therapy subgroup (RR 1.16, 95% CI 0.91-1.48; *P*=.23; Figure 2C). In addition, compared to the control group, individuals aged ≤60 years had higher *H*. *pylori* eradication rates after NME (RR 1.21, 95% CI 1.02-1.44; *P*=.03), whereas those aged >60 years did not (RR 1.21, 95% CI 0.91-1.60; *P*=.18; Figure 3A [20-32]). Interestingly, individuals from low- and middle-income countries showed significantly increased *H*. *pylori* eradication rates after NME compared to the control group (RR 1.24, 95% CI 1.13-1.37; *P*<.001), whereas there was no impact on individuals from high-income countries (RR 1.08, 95% CI 0.79-1.50; *P*=.62; Figure 3B). Regardless of whether routine outpatient *H*. *pylori* eradication education was provided, NME yielded higher eradication rates in both the traditional (RR 1.22, 95% CI 1.03-1.43; *P*=.02) and nontraditional (RR 1.22, 95% CI 1.08-1.37; *P*=.002; Figure 3C) education subgroup. Finally, using WeChat as a new media platform for *H*. *pylori* education, there was no significant difference in *H*. *pylori* eradication rates between the NME group and the control group when the WeChat group functionality was used (RR 1.21, 95% CI 0.93-1.58; *P*=.16; Figure 4A [21-25]). However, when using WeChat for one-on-one education or other functionalities, the NME group showed higher *H*. *pylori* eradication rates (RR 1.31, 95% CI 1.08-1.59; *P*=.006; Figure 4A). When the treatment duration was 14 days (RR 1.26, 95% CI 1.14-1.39; *P*<.001), the *H*. *pylori* eradication rate in the NME group was consistently higher than that in the control group (Figure S2A in Multimedia Appendix 2). However, when the duration was less than 14 days (RR 1.06, 95% CI 0.85-1.32; *P*=.61), there was no significant difference in eradication rates between the 2 groups (Figure S2A in Multimedia Appendix 2).

#### Patient Compliance

**Figure 5:**
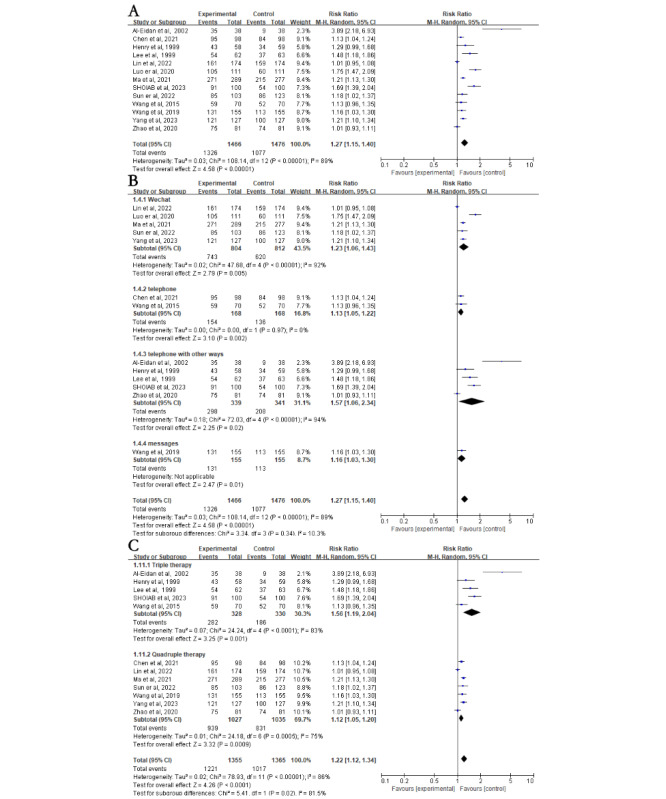
Forest plot comparing patient compliance in education versus control groups—subgroup analyses on intervention methods and regimens. (A) Forest plot comparing patient compliance between the education and control groups. (B) Subgroup analysis for patient compliance based on different intervention methods. (C) Subgroup analysis for patient compliance based on different eradication regimens. Control: traditional instructions; experimental: new media–based education instructions.

All 13 studies, involving 2942 patients, reported data on patient adherence. The analysis showed that patients in the NME group had significantly higher medication adherence compared to those in the control group (90.5% vs 73%; RR 1.27, 95% CI 1.15-1.40; *P*<.001; Figure 5A [20-32]). There was high statistical heterogeneity between studies (*I*^2^=89%; *P*<.001), whereas sensitivity analysis showed stable results (Figure 5A).

#### Subgroup Analysis of Compliance

**Figure 6:**
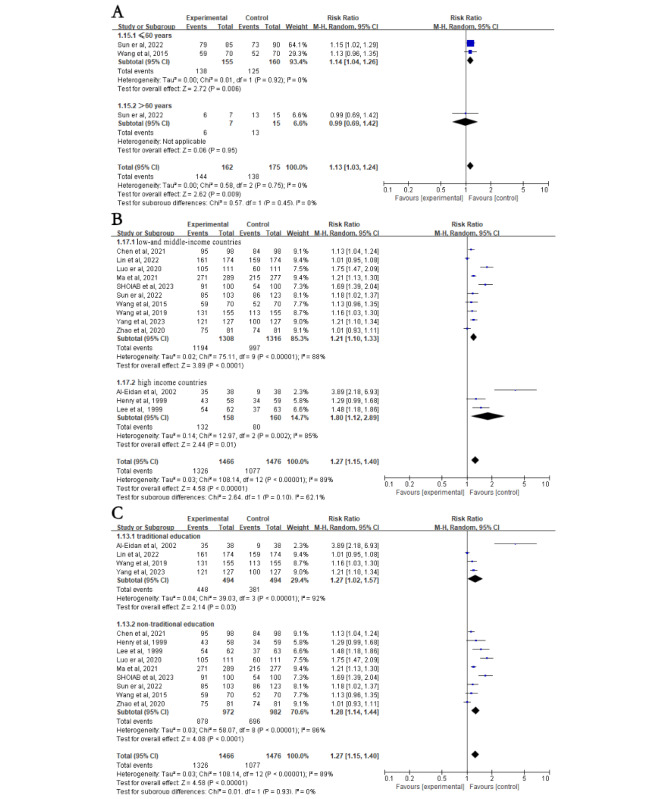
Subgroup analysis on patient compliance based on age (>60 years), country socioeconomic status (high income vs low and middle income), and education (traditional). (A) Subgroup analysis for patient compliance based on whether patients were older than 60 years. (B) Subgroup analysis for patient compliance based on whether patients were from high-income or low- and middle-income countries. (C) Subgroup analysis for patient compliance based on whether all patients received traditional education. Control: traditional instructions; experimental: new media–based education instructions.

A stratified analysis based on different NME methods and different medication regimens showed that the NME group clearly had better compliance than the control group using WeChat (RR 1.23, 95% CI 1.06-1.43; *P*=.005), telephone (RR 1.13, 95% CI 1.05-1.22; *P*=.002), telephone with other methods (RR 1.57, 95% CI 1.06-2.34; *P*=.02), SMS text messaging (RR 1.16, 95% CI 1.03-1.30; *P*=.01; Figure 5B), triple therapy regimen (RR 1.56, 95% CI 1.19-2.04; *P*=.001), or quadruple therapy regimen (RR 1.12, 95% CI 1.05-1.20; *P*<.001; Figure 5C). A stratified analysis based on patient age showed that NME group clearly had better compliance than the control group in the group aged ≤60 years (RR 1.14, 95% CI 1.04-1.26; *P*=.006) but not in the group aged >60 years (RR 0.99, 95% CI 0.69-1.42; *P*=.95; Figure 6A). Another stratified analysis based on whether the patients were from high-income countries showed that the NME group clearly had better compliance than the control group in both the low- and middle-income country subgroup (RR 1.21, 95% CI 1.10-1.33; *P*<.001) and the high-income country subgroup (RR 1.80, 95% CI 1.12-2.89; *P*=.01; Figure 6B [20-32]). Similarly, based on whether basic outpatient education was provided to all enrolled patients, the NME group clearly had better compliance than the control group in both the traditional education subgroup (RR 1.27, 95% CI 1.02-1.57; *P*=.03) and the nontraditional education subgroup (RR 1.28, 95% CI 1.14-1.44; *P*<.001; Figure 6C). A stratified analysis based on whether WeChat group re-education was used showed that new media significantly increased patient compliance in the non-WeChat subgroup (RR 1.31, 95% CI 1.08-1.59; *P*=.009) but not in the WeChat subgroup (RR 1.21, 95% CI 0.93-1.58; *P*=.28; Figure 4B). In the subgroup with a 14-day treatment duration, patient compliance was significantly higher in the NME group than in the control group (RR 1.24, 95% CI 1.12-1.37; *P*<.001; Figure S2B in Multimedia Appendix 2). In contrast, in regimens lasting less than 14 days, compliance did not differ significantly between the 2 groups (RR 1.65, 95% CI 0.97-2.82; *P*=.07; Figure S2B in Multimedia Appendix 2).

### Secondary Outcomes

#### Adverse Events

Adverse drug reactions were documented in 69% (9/13) of the studies. The primary adverse events reported included diarrhea, constipation, nausea, abdominal pain, taste disorder, and skin rash. No significant differences were found between the NME and control groups (RR 0.88, 95% CI 0.71-1.09; *P*=.23; Figure S3A in Multimedia Appendix 2). Although significant heterogeneity was present among the studies (*I*^2^=63%; *P*<.001), the findings remained stable in sensitivity analysis (Figure S3A in Multimedia Appendix 2).

#### Patient Satisfaction

A total of 23% (3/13) of the studies, involving 672 patients, provided data on patient satisfaction. Patient satisfaction in the NME group was notably higher than that in the control group (61% vs 47.7%; RR 1.30, 95% CI 1.13-1.49; *P*<.001). Low statistical heterogeneity was observed (*I*^2^=0%; *P*=.47; Figure S3B in Multimedia Appendix 2).

### Sensitivity and Publication Bias Analyses

The results of this study revealed some statistical heterogeneity, which may be attributed to factors such as intervention frequency, intervention duration, patient source (ie, patients from high-income vs low- and middle-income countries), and variation in eradication regimens. Given that heterogeneity is a major concern in meta-analysis, we conducted sensitivity analyses by sequentially excluding individual studies. The pooled results for all 4 main outcomes (*H*. *pylori* eradication rate, patient compliance, adverse events, and patient satisfaction) remained stable. In addition, subgroup analyses were conducted according to intervention type, treatment regimen, age group, country socioeconomic status, provision of baseline outpatient education, and use of the WeChat group functionality.

For publication bias, we first conducted visual inspection of funnel plots and then applied the Egger linear regression test and Begg rank correlation test for the 2 primary outcomes (*H*. *pylori* eradication rate and patient compliance) as both included ≥10 studies. Although mild asymmetry was observed in the funnel plots upon visual inspection (Figure S4 in Multimedia Appendix 2), statistical tests did not suggest significant publication bias. For *H*. *pylori* eradication rate, the Egger test resulted in *P*=.93, whereas the Begg test showed a Kendall tau with continuity correction of −0.05 (*P*=.84). Similarly, for patient compliance, the Egger test resulted in *P*=.20, and the Begg test reported a Kendall tau with continuity correction of 0.32 (*P*=.13). These findings indicate a low likelihood of publication bias; however, the slight visual asymmetry warrants cautious interpretation of the results.

## Discussion

### Principal Findings

This meta-analysis delved into the impact of NME on *H*. *pylori* eradication rates using platforms such as the internet or mobile communication. Overall, NME exhibited superiority over traditional education in both *H*. *pylori* eradication rates and patient compliance. Specifically, interventions via WeChat demonstrated clear advantages over telephone and SMS text messaging interventions. However, in comparison to traditional education, NME did not significantly ameliorate the overall occurrence rate or specific adverse reactions.

Compared with previous meta-analyses [17-19], our study included a larger and more recent set of trials (13 studies up to February 2025), thereby enhancing statistical power and ensuring that the evidence reflected the latest data. Moreover, we conducted more comprehensive subgroup analyses, not only covering intervention type, treatment regimen, patient satisfaction, and adverse events but also further exploring patient age, country socioeconomic status, provision of baseline outpatient education, and WeChat intervention models (personalized one-on-one vs group based). These additional analyses allowed us to better explain heterogeneity and provide novel insights into the applicability and optimization of NME strategies across different populations and social contexts.

Patient compliance stands as a pivotal factor contributing to the poor eradication rate of *H*. *pylori*. Adequate compliance is pivotal for ensuring the correct implementation of any eradication program, whereas poor compliance often leads to failure [11,35]. Therefore, educational interventions aimed at enhancing patient compliance are essential. Particularly with quadruple therapy, which yields better *H*. *pylori* eradication effects than triple therapy but entails a broader spectrum of drugs and necessitates more frequent dosing, patients may easily overlook or miss doses [13]. Thus, based on the findings of this study, NME significantly bolsters *H*. *pylori* eradication rates in the quadruple therapy population.

Compared to other strategies to enhance eradication rates, such as selecting antibiotics based on drug susceptibility, educational interventions are deemed cost-effective and straightforward [8]. Furthermore, education can augment patient awareness of *H*. *pylori*, foster self-management, and facilitate physician-patient communication [36]. NME holds even more promise than traditional education methods as it equips patients to better comprehend treatment regimens, provides convenient information on adverse drug reactions and health knowledge, and offers more interactive solutions to patients’ issues [22]. Particularly in low- and middle-income countries, where the average educational attainment level may not match that of high-income nations, repetitive education via new media can significantly enhance understanding and, thereby, improve *H*. *pylori* eradication rates.

The intervention measures used in the studies included in this review encompassed telephone follow-ups, SMS text message reminders, and WeChat consultations. WeChat interventions were used in 38% (5/13) of the studies, all of which showcased its effectiveness in enhancing the eradication rate. WeChat serves as a valuable platform for timely communication between patients and physicians, facilitating the prompt resolution of medication-related issues.

It is pertinent to note the limitations of emerging technologies such as WeChat. The inference drawn from this study is that older individuals, who are less inclined to adopt electronic devices, may not derive as much benefit from WeChat software education. Conversely, younger and middle-aged individuals exhibit greater receptiveness to new technologies, thereby perfectly exemplifying the advantages of WeChat education. Notably, our findings suggest that education delivered through WeChat groups may be less effective compared to one-on-one interactions. This difference may be attributed to several factors. First, group-based education often lacks personalization and may dilute individual responsibility, leading to lower engagement. Second, patients may be reluctant to discuss personal health issues in a group setting due to perceived privacy concerns. Third, the passive nature of group messages can result in reduced attentiveness and limited interaction. In contrast, stand-alone applications or supervised one-on-one education offer more tailored support, thereby enhancing compliance and *H*. *pylori* eradication rates. Due to the absence of direct comparative studies across different NME delivery modes, the most effective approach or combination of methods remains uncertain.

The internet and smartphones have revolutionized information access, and mobile health apps can effectively deliver patient education [14]. Given the exponential growth of medical apps, evaluating their capacity to provide comprehensive health education is imperative [15].

Enhancing patient compliance is not associated with potential adverse events. Given that treatment regimens usually incorporate antibiotics, side effects are often attributed to antibiotic-related adverse events [37]. However, different treatment schemes yield varying side effects, underscoring the significance of precisely defining adverse events in this context [38]. While fortified NME can mitigate adverse emotions and mild discomfort stemming from side effects through enhanced physician-patient communication, its efficacy in reducing the occurrence rate of side effects remains limited.

Given the limitations of certain studies, the findings of this review warrant cautious interpretation. Primarily, most studies (10/13, 77%) were conducted in Asia, with only 23% (3/13) originating from Western countries. This underscores the necessity for trials encompassing diverse ethnic groups as the *H*. *pylori* infection rate varies across geographic regions [13]. Consequently, prudence should be exercised when extrapolating the conclusions to other regions. Second, the overall sample size was relatively modest. The absence of multicenter and large-sample studies amplifies the potential for bias and error, underscoring the need for additional rigorously designed experiments. Third, the interventions spanned a spectrum across the study sample, rendering it challenging to draw overarching conclusions. Meanwhile, although this review primarily relied on RCTs, it also included 2 retrospective cohort studies. Despite a certain level of comparability in terms of intervention content and study quality, it is important to note that differences in study design may introduce additional systematic bias, thereby weakening the strength of the conclusions. Finally, all fortified education methods engendered a selection bias toward specific demographic groups. For instance, WeChat education may not be viable for patients without smartphones. Consequently, these intervention measures may not fully capture patients with diverse educational backgrounds and economic statuses. Therefore, future research should prioritize the inclusion of more high-quality RCTs while also expanding intervention approaches tailored to diverse populations to enhance the external validity and generalizability of the findings.

### Conclusions

In summary, the findings suggest that NME holds promise in increasing the *H*. *pylori* eradication rate, particularly when implemented via mobile phone apps. Nevertheless, intervention methods and designs exhibit considerable heterogeneity, necessitating ongoing high-quality research. Furthermore, the evolution of artificial intelligence technology has shifted the focus of medical and health education from the populace to the individual level. Hence, deliberating on the creation of personalized education programs and their integration into clinical practice is imperative.

## Data Availability

The datasets generated or analyzed during this study are available from the corresponding author on reasonable request.

## Appendix

Multimedia Appendix 1 Detailed search strategies.

Multimedia Appendix 2 Supplementary materials and visual abstract.

Multimedia Appendix 3 PRISMA checklist.

## Abbreviations

ITT: intention-to-treat
MeSH: Medical Subject Headings
NME: new media–based education
PP: per-protocol
PRISMA: Preferred Reporting Items for Systematic Reviews and Meta-Analyses
RCT: randomized controlled trial
RoB 2: Cochrane risk-of-bias tool for randomized trials
ROBINS-I: Risk of Bias in Nonrandomized Studies of Interventions
RR: risk ratio
