# Supplementary material for: Effects of New Media–Based Education on the Treatment of Helicobacter pylori Infection: Systematic Review and Meta-Analysis
Source: J Med Internet Res. 2025 Oct 23;27:e78387. doi: 10.2196/78387 (PMC12592897; doi:10.2196/78387)
Supplement: Multimedia Appendix 1 [file jmir_v27i1e78387_app1.doc]

| **Database** | **Search Strategy** |
| --- | --- |
| PubMed | (((“Helicobacter pylori”[Mesh] OR “Helicobacter pylori”[tiab] OR “H. pylori”[tiab])) AND ((“patient education as topic”[Mesh] OR “patient education”[tiab] OR “health education”[tiab] OR “educational technology”[tiab] OR “mobile phone”[tiab] OR “cell phone”[tiab] OR “smartphone”[tiab] OR “text message*”[tiab] OR “short message service”[tiab] OR “SMS”[tiab] OR “WeChat”[tiab] OR “tele-education”[tiab] OR “telemedicine”[tiab] OR “digital health”[tiab]))) AND ((randomized controlled trial[pt] OR controlled clinical trial[pt] OR cohort studies[Mesh] OR prospective studies[Mesh] OR retrospective studies[Mesh])) |
| EMBASE (Elsevier) | (‘helicobacter pylori’/exp OR ‘helicobacter pylori’:ab,ti OR ‘h pylori’:ab,ti) AND (‘patient education’/exp OR ‘health education’:ab,ti OR ‘educational technology’:ab,ti OR ‘mobile phone’/exp OR ‘cell phone’:ab,ti OR ‘smartphone’:ab,ti OR ‘text message’/exp OR sms:ab,ti OR wechat:ab,ti OR ‘tele-education’:ab,ti OR ‘telemedicine’/exp OR ‘digital health’:ab,ti) AND (‘randomized controlled trial’/exp OR ‘controlled clinical trial’/exp OR ‘cohort analysis’/exp OR ‘prospective study’/exp OR ‘retrospective study’/exp) |
| Web of Science (Core Collection) | TS = ((“Helicobacter pylori” OR “H. pylori”) AND (“patient education” OR “health education” OR “educational technology” OR “mobile phone” OR “cell phone” OR “smartphone” OR “text message*” OR “SMS” OR “WeChat” OR “tele-education” OR “telemedicine” OR “digital health”)) AND TS = ((randomized controlled trial) OR (controlled clinical trial) OR (cohort study) OR (prospective study) OR (retrospective study)) |
| Cochrane Library (CENTRAL) | (“Helicobacter pylori” OR “H. pylori”): ti,ab,kw AND (“patient education” OR “health education” OR “educational technology” OR “mobile phone” OR “cell phone” OR “smartphone” OR “text message*” OR “SMS” OR “WeChat” OR “tele-education” OR “telemedicine” OR “digital health”):ti,ab,kw |

**Multimedia Appendix 1.** Detailed Search Strategies
