# Supplementary material for: Effects of New Media–Based Education on the Treatment of Helicobacter pylori Infection: Systematic Review and Meta-Analysis
Source: J Med Internet Res. 2025 Oct 23;27:e78387. doi: 10.2196/78387 (PMC12592897; doi:10.2196/78387)
Supplement: Multimedia Appendix 2 [file jmir_v27i1e78387_app2.pdf]

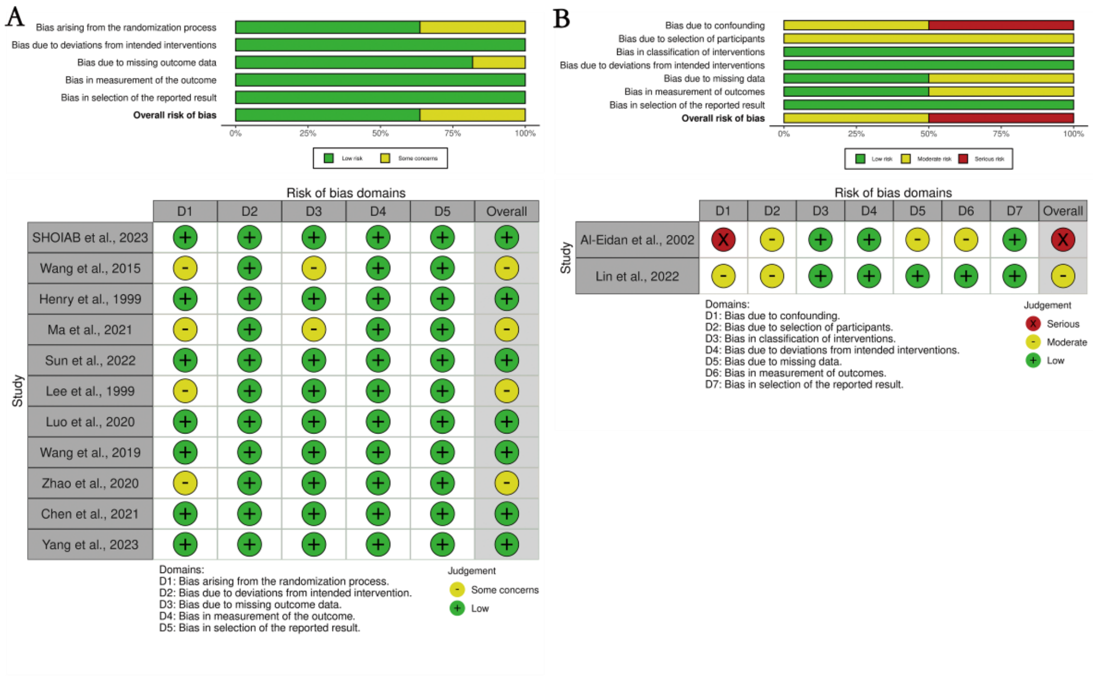

Supplementary Figure 1. Quality assessment results of included studies. (A) Risk of bias assessment for randomized controlled trials using the Cochrane Risk of Bias 2 (RoB 2) tool. (B) Risk of bias assessment for retrospective cohort studies using the ROBINS-I tool.

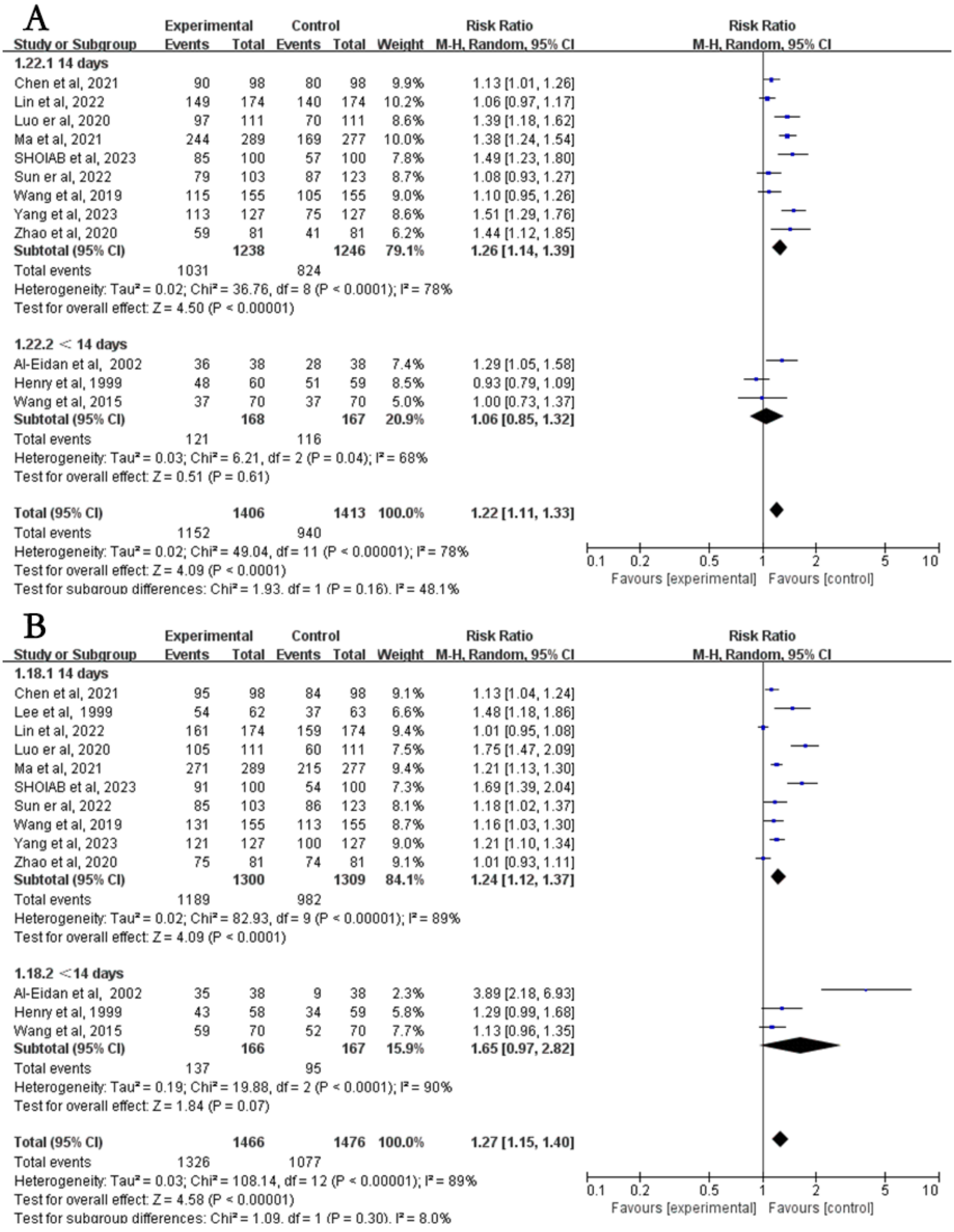

Supplementary Figure 2. Subgroup analysis of *H. pylori* eradication rate and patient compliance based on different treatment durations. (A) Subgroup analysis for *H. pylori* eradication rate. (B) Subgroup analysis for patient compliance

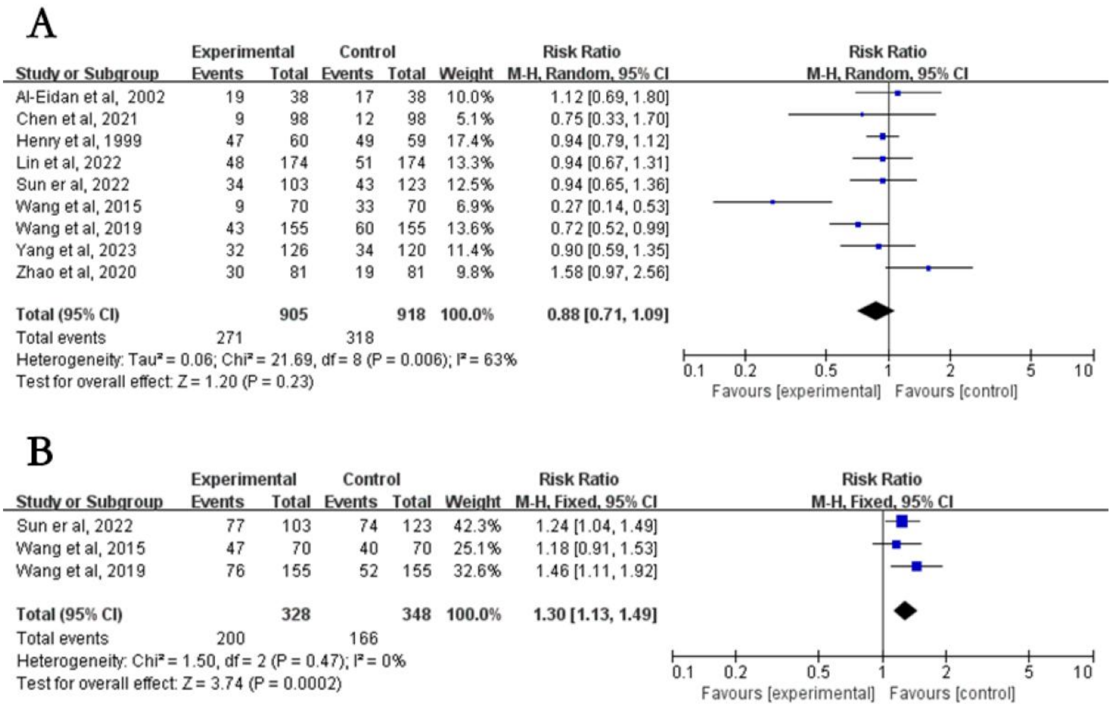

Supplementary Figure 3. (A) Forest plot comparing adverse events between educational groups and control groups; (B) Forest plot comparing satisfaction between educational groups and control groups.

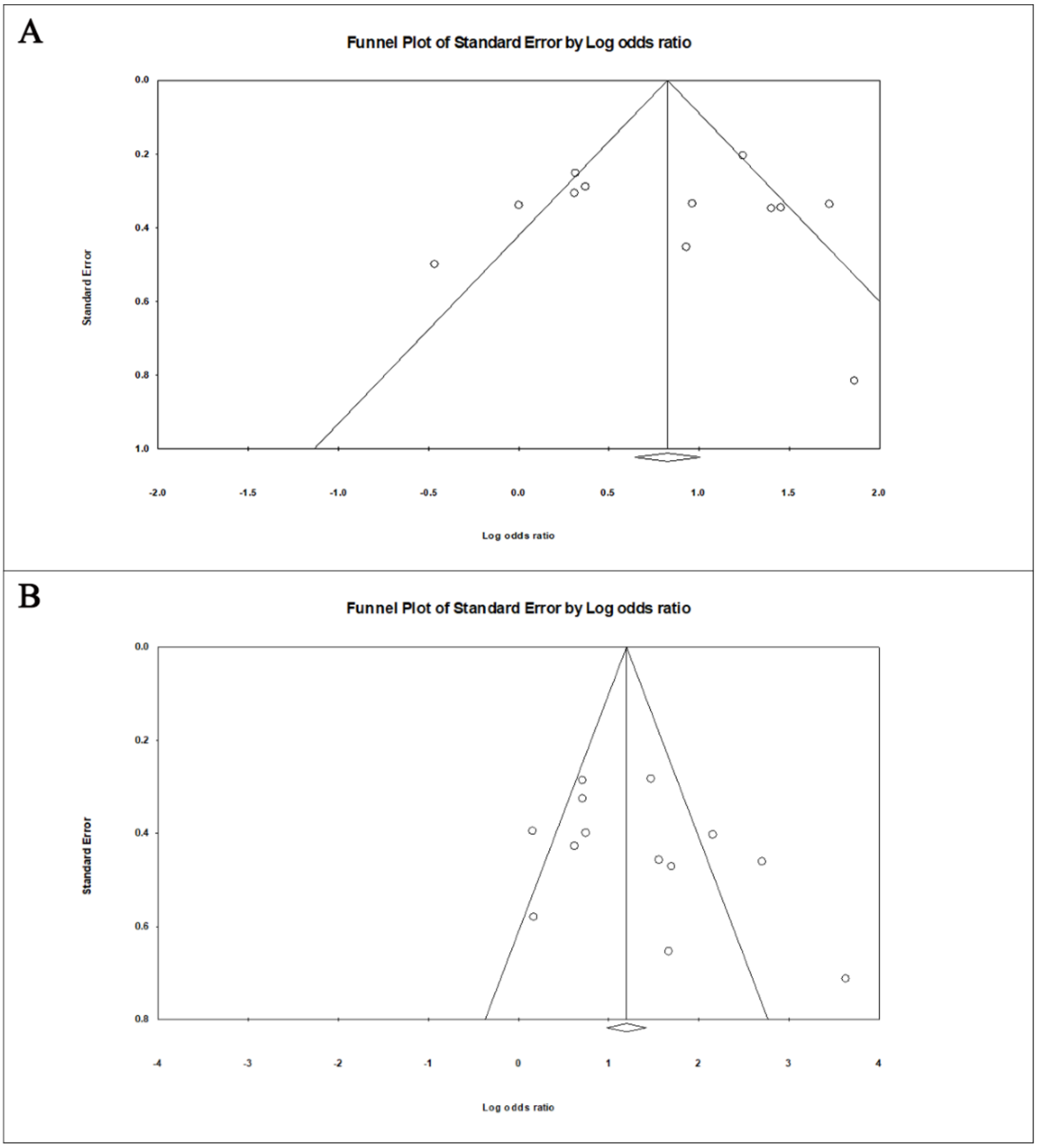

Supplementary Figure 4. Funnel plots assessing publication bias of included studies.

(A) Funnel plot for *H. pylori* eradication rate. (B) Funnel plot for patient compliance.

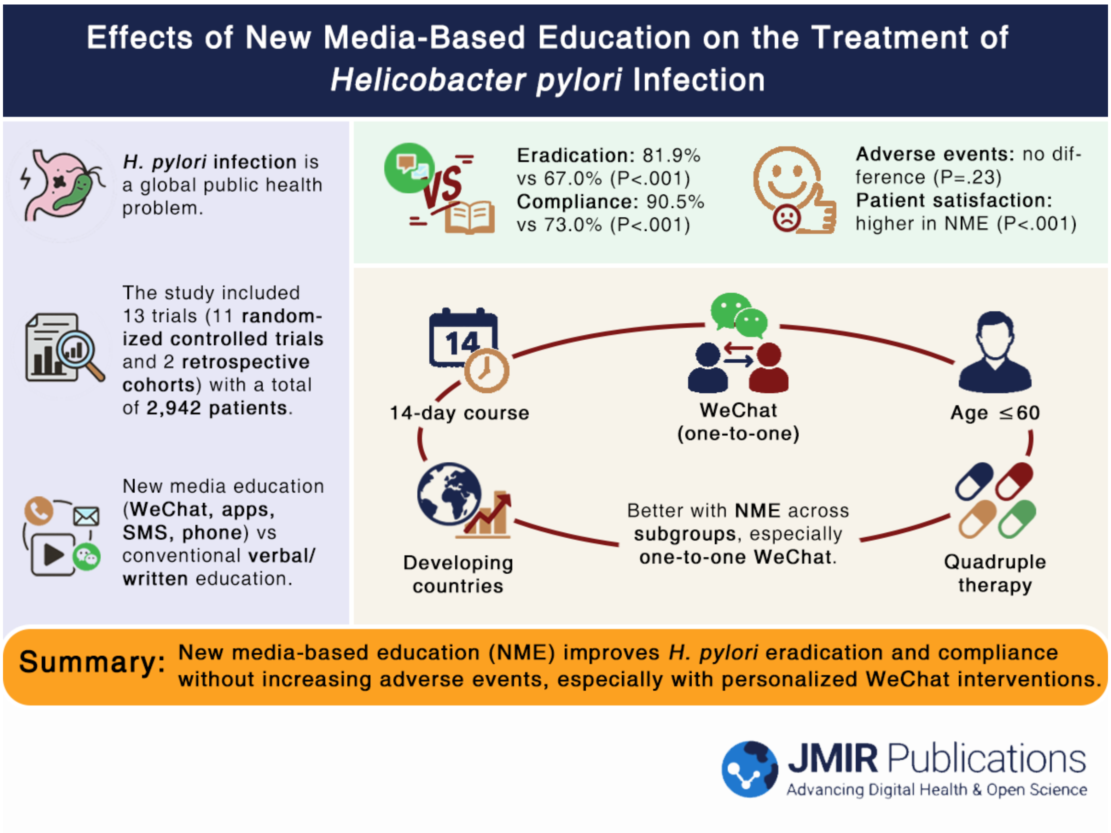

Visual Abstract. New media–based education improves *Helicobacter pylori* eradication and treatment adherence.
